# Identification of Key Genes Involved in Pancreatic Ductal Adenocarcinoma with Diabetes Mellitus Based on Gene Expression Profiling Analysis

**DOI:** 10.3389/pore.2021.604730

**Published:** 2021-04-20

**Authors:** Weiyu Zhou, Yujing Wang, Hongmei Gao, Ying Jia, Yuanxin Xu, Xiaojing Wan, Zhiying Zhang, Haiqiao Yu, Shuang Yan

**Affiliations:** ^1^ Department of Endocrinology, The Fourth Affiliated Hospital of Harbin Medical University, Harbin, China; ^2^ Department of Neurology, The Fourth Affiliated Hospital of Harbin Medical University, Harbin, China; ^3^ Department of Pathology, The First Affiliated Hospital of Harbin Medical University, Harbin, China

**Keywords:** diabetes mellitus, pancreatic ductal adenocarcinoma, meta-analysis, support vector machine, survival analysis

## Abstract

This study aimed to identify key genes involved in the progression of diabetic pancreatic ductal adenocarcinoma (PDAC). Two gene expression datasets (GSE74629 and GSE15932) were obtained from Gene Expression Omnibus. Then, differentially expressed genes (DEGs) between diabetic PDAC and non-diabetic PDAC were identified, followed by a functional analysis. Subsequently, gene modules related to DM were extracted by weighed gene co-expression network analysis. The protein-protein interaction (PPI) network for genes in significant modules was constructed and functional analyses were also performed. After that, the optimal feature genes were screened by support vector machine (SVM) recursive feature elimination and SVM classification model was built. Finally, survival analysis was conducted to identify prognostic genes. The correlations between prognostic genes and other clinical factors were also analyzed. Totally, 1546 DEGs with consistent change tendencies were identified and functional analyses showed they were strongly correlated with metabolic pathways. Furthermore, there were two significant gene modules, in which RPS27A and UBA52 were key genes. Functional analysis of genes in two gene modules revealed that these genes primarily participated in oxidative phosphorylation pathway. Additionally, 21 feature genes were closely related with diabetic PDAC and the corresponding SVM classifier markedly distinguished diabetic PDAC from non-diabetic PDAC patients. Finally, decreased KIF22 and PYGL levels had good survival outcomes for PDAC. Four genes (RPS27A, UBA52, KIF22 and PYGL) might be involved in the pathogenesis of diabetic PDAC. Furthermore, KIF22 and PYGL acted as prognostic biomarkers for diabetic PDAC.

## Introduction

Pancreatic cancer (PC) is the third leading cause of cancer-associated mortality around the world. Compelling evidence has suggested that PC is dominated by pancreatic ductal adenocarcinoma (PDAC) which accounts for approximately 95% of PC, and PDAC is an aggressive tumor with high incidence and metastasis rates [1, 2]. It is reported that dietary factors are primary causes for carcinogenesis [3]. Moreover, numerous epidemiological and cohort studies have indicate that diabetes mellitus (DM) is a risk factor for PDAC progression [4, 5]. Huxley *et al* found that patients diagnosed with DM (<4 years) have 50% higher risk of PC than those who suffered from DM ≥ 5 years [6]. Kleeff *et al* evaluated the correlations between clinical factors and DM in patients with PC, and they observed that diabetic patients who received PC resection and adjuvant therapy had a larger tumor size and a higher death risk than non-diabetic patients [7]. Moreover, the new-onset DM is predominately correlated with early recurrence rate in PC patients undergoing resection, implying new-onset DM might be an important clinical manifestation for PC and new-onset DM detection might be helpful for early diagnosis for PC [8]. Additionally, many researchers have also argued that PDAC could cause DM, such as type 3C diabetes [9]. Therefore, the underlying association between PDAC and DM is complicated due to the presence of a bidirectional link.

Encouragingly, a growing number of studies have focused on exploring the underlying molecular mechanisms of diabetic PDAC. Sun *et al* noted that transgelin-2 encoded by *TAGLN2* was significantly up-regulated in PDAC tissues and in a subgroup of PDAC patients suffering from DM, suggesting transgelin-2 was possibly implicated in the development of DM coexisting with PDAC [10]. Boursi *et al* constructed a clinical prediction model based on several risk factors for DM to evaluate PC risk among those individuals with new-onset diabetes [11]. Besides, an early research demonstrated that the expression levels of *VNN1* and *MMP9* were elevated in patients with PC-associated DM and these two genes could well discriminate PC-related DM from type 2 diabetes by using a microarray analysis [12]. Later on, investigators found that *VNN1* overexpression in PC-associated new-onset DM aggravated paraneoplastic islet dysfunction by the increase of oxidative stress base on the laboratory research [13]. However, an integrated analysis for identifying the potential biomarkers involved in diabetic PDAC has not been performed.

Therefore, we conducted an integrated meta-analysis for gene expression profiles of diabetic PDAC to screen novel therapeutic targets for diabetic PDAC. Differentially expressed genes (DEGs) were firstly identified between diabetic PDAC and non-diabetic PDAC patients. Then, functional analyses were conducted to explore the underlying roles of DM-related genes on PDAC progression. Finally, prognosis-associated genes were further extracted by survival analysis.

## Materials and Methods

### Data Acquisition and Pre-processing

Gene expression datasets were searched with the keywords of “pancreatic adenocarcinoma,” “diabetes” and “homo sapiens”, and eligible datasets were downloaded from Gene Expression Omnibus (GEO; http://www.ncbi.nlm.nih.gov/geo/) database [14]. The selection criteria for microarray datasets were as follows: 1) whole genome expression data of peripheral blood samples; 2) all samples with relevant DM information; and 3) dataset with the sample size not less than 15. Consequently, there were two datasets (GSE74629 and GSE15932) available after dataset screening. The platform for GSE74629 was Illumina HumanHT-12 V4.0 expression beadchip, which was comprised of 36 samples from PDAC patients (14 patients with diabetes and 22 patients without diabetes). Raw TXT files of GSE74629 were obtained and probes were converted into gene symbols by using the platform annotation files. When multiple probes were mapped to the same gene symbol, average value of different probes was considered as the final gene expression level. For GSE15932, there were 16 samples from PDAC patients, among whom there were 8 PDAC patients complicated with diabetes and 8 PDAC patients not complicated with diabetes. GSE15932 was based on the Affymetrix Human Genome U133 Plus 2.0 Array platform and the original Affymetrix CEL files were downloaded. Raw data of these two datasets were pre-processed with oligo [16] package (Version 3.6; http://www.bioconductor.org/packages/release/bioc/html/oligo.html) in R 3.4.1, including imputing missing data with median values, background correction by using MicroArray Suite (MAS) method, and quantile normalization. Then, gene expression values were subjected to log_2_ transformation with Limma (Version 3.34.0; https://bioconductor.org/packages/release/bioc/html/limma.html) package [15] in R 3.4.1 to ensure normal distribution.

### DEGs Identification and Functional Analyses

MetaDE package (https://cran.r-project.org/web/packages/MetaDE) in R 3.4.1 was utilized to eliminate statistical deflection for integrating two datasets from different sources [17]. Briefly, heterogeneity test of each gene expression value in different platform was firstly performed based on three parameters (tau^2^, Q value, and Q pval). Generally, subjects were considered as homogenous when tau^2^ was 0. Meanwhile, when Q value was subjected to chi-square test with K-1 freedom and Q pval value was greater than 0.05, the study subjects was also homogeneous without bias. Then, gene expression differences between DM and non-DM group in integrated dataset were estimated by *p* value, which was further adjusted into false discovery rate (FDR) by algorithm in MetaDE package. The tau^2^ = 0 and Q pval >0.05 were set as thresholds of homogeneous test, and genes with FDR <0.05 was regarded as significantly differentially expressed in inter-group comparison. According to the calculated log_2_fold change (FC), DEGs with consistent expression patterns in two datasets were remained for the following functional and pathway enrichment analyses. Database for Annotation Visualization and Integrated Discovery (DAVID) consists of an integrated biological knowledgebase and analytic tools that aimed at systematically extracting biological meaning from large gene/protein lists [18, 19]. Thus, DAVID (Version 6.8, https://david.ncifcrf.gov/) was employed to conduct Gene Ontology-biological process (GO-BP) and Kyoto Encyclopedia of Genes and Genomes (KEGG) pathway enrichment analyses, and *p* value <0.05 was used as the cutoff level of significant enrichment.

### Co-Expression Module Analysis

Weighed gene co-expression network analysis (WGCNA) has been successfully applied in discovery of interest modules and identification of key genes in modules. GSE74629 with a larger sample size was acted as a training dataset and GSE15932 was considered as a verification dataset in this study. We used WGCNA (Version 1.61; https://cran.r-project.org/web/packages/WGCNA/index.html) to extract the significantly extracted stable gene modules related to DM [20]. The thresholds of gene modules screening were set as the number of genes in modules ≥80 and cutHeight = 0.995.

### Protein-Protein Interaction (PPI) Network Analysis

Protein-protein interactions of genes in significant modules were revealed by Search Tool for the Retrieval of Interacting Genes (STRING) [21] database (Version 10.0; https://string-db.org/), which provides a critical assessment and integration of protein-protein interactions. The revealed protein-protein interactions were used to construct PPI network which was visualized with Cytoscape (version 3.6.1; http://www.cytoscape.org/) [22]. In addition, functional analyses (the GO-BP analysis and KEGG pathway analysis) of genes in constructed PPI network were carried out by using DAVID (Version 6.8, https://david.ncifcrf.gov/) [18, 19], with the cut-off threshold of *p* value <0.05.

### Optimization of Feature Genes and SVM Classifier Construction

Support vector machine recursive feature elimination (SVM-RFE) is a feature-selection method by iteratively ranking features and removing the lowest features [23]. Herein, GSE74629 was used as the training dataset while GSE15932 served as the validation dataset. We employed the RFE algorithm of caret package (Version 6.0-76; https://cran.r-project.org/web/packages/caret) in R 3.4.1 to identify the optimal feature gene set which had the highest accuracy in 10-fold cross validation test [24]. To further extract the key genes involved in diabetic DM, a SVM-based classifier was built by SVM method of e1071 package (version 1.6-8; https://cran.r-project.org/web/packages/e1071) in R 3.4.1 with optimal feature genes based on core of sigmoid kernel and 100-fold cross validation [25]. Furthermore, performance evaluation of SVM classifier was performed in training and validation datasets, respectively. Receiver operating characteristic (ROC) curve was constructed and the area under ROC (AUROC) value was calculated. Here, the pROC package (version 1.12.1; https://cran.r-project.org/web/packages/pROC/index.html) in R 3.4.1 was used to calculate several indicators for ROC curve, including sensitivity (Sen), specificity (Spe), positive predictive value (PPV) and negative predictive value (NPV) \ Sensitivity = true positive/(true positive + false negative); Specificity = true negative/(false positive + true negative); PPV = true positive/(true positive + false positive); NPV = true negative/(true negative + false negative) [26].

### Survival Analysis

Public gene expression profiles of PC were downloaded from The Cancer Genome Atlas (TCGA) database, and gene expression profiles of 150 patients with PC were obtained. Gene expression profiles were measured experimentally with the Illumina HiSeq 2000 RNA Sequencing platform by the University of North Carolina TCGA genome characterization center. Level 3 data was downloaded from TCGA data coordination center. This dataset shows the gene-level transcription estimates, as in log2(x+1) transformed RSEM normalized count. Meanwhile, the corresponding clinical information was also downloaded and obtained. There were 114 patients had clinical information about DM, including 33 diabetic PDAC patients and 81 non-diabetic PDAC patients. In order to identify prognostic genes, univariate cox regression analysis was performed between features gene in SVM classifier and clinical survival by survival (Version 2.41-1; http://bioconductor.org/packages/survivalr/) package in R 3.4.1 [27]. All the 114 samples were divided into high and low risk groups based on the expression levels of prognostic genes, with the cutoff threshold of median expression level calculated from all the 114 samples (high risk group: expression level > median expression level; low risk group: expression level < median expression level). Survival analysis was conducted and Kaplan-Meier (KM) survival curves were generated. Finally, univariate cox regression analyses of prognostic genes with other clinical parameters (age, gender, history of chronic pancreatitis, history of diabetes, alcohol history, neoplasm_histologic grade, pathologic M, pathologic N, pathologic T, and pathologic stage) were performed by using glm function in R software.

## Results

### DEGs Screening and Functional Analyses

**FIGURE 1:**
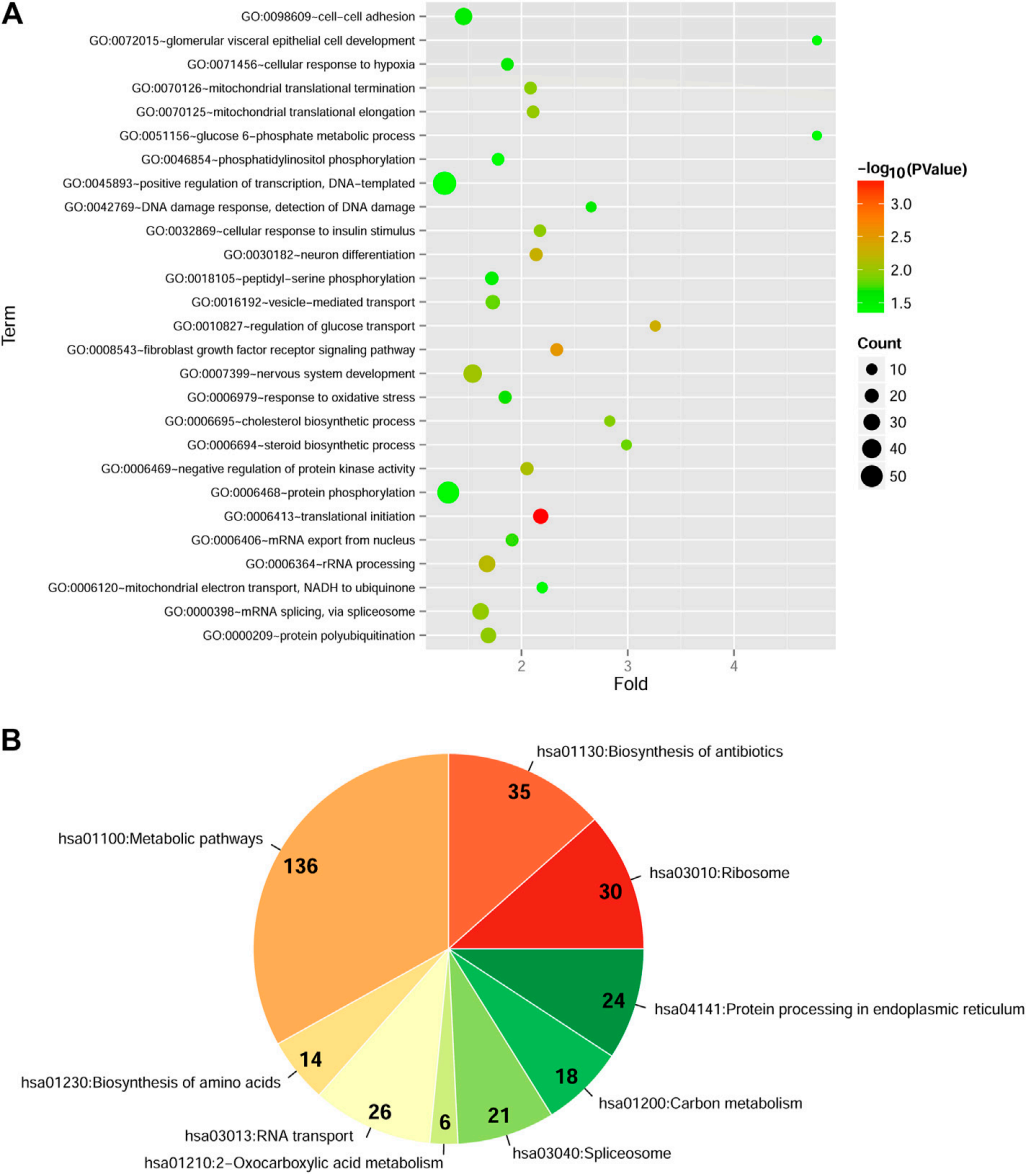
Functional and pathway enrichment analyses of differentially expressed genes (DEGs) with consistent change patterns between GSE74629 and GSE15932 **(A)**: The significantly enriched Gene Ontology-biological process (GO-BP) terms. Vertical axis shows enrichment fold values and horizontal axis shows the names of GO-BP terms. Node size denotes the number of genes, and the bigger node the larger number of genes. Node color indicates the enrichment significance, the closer to the red the higher significance **(B)**: The significantly enriched Kyoto Encyclopedia of Genes and Genomes (KEGG) pathways. The parts in pie chart represent the specific KEGG pathways. The Arabic numerals show the number of DEGs involved in each KEGG pathway. The color indicates the enrichment significance, the closer to the red the higher significance.

After data pre-processing of two datasets, a total of 1546 DEGs with consistent change patterns between diabetic PDAC patients and non-diabetic PDAC patients were uncovered. Moreover, bidirectional hierarchical clustering analysis showed that these genes were dramatically differentially expressed and their differential expressions were consistent in each dataset, implying that they had similar expression pattern in two datasets (Supplementary Figure S1). Additionally, GO-BP analysis indicated that these DEGs were significantly enriched in 27 GO-BP terms, such as translational initiation, fibroblast growth factor receptor signaling pathway and regulation of glucose transport (Figure 1A, Supplementary Table S1). Simultaneously, there were 9 markedly enriched KEGG pathways for these DEGs and vast majority of these DEGs were mainly involved in metabolic pathways (Figure 1B, Supplementary Table S1).

### Identification of Significant Gene Modules by WGCNA

**FIGURE 2:**
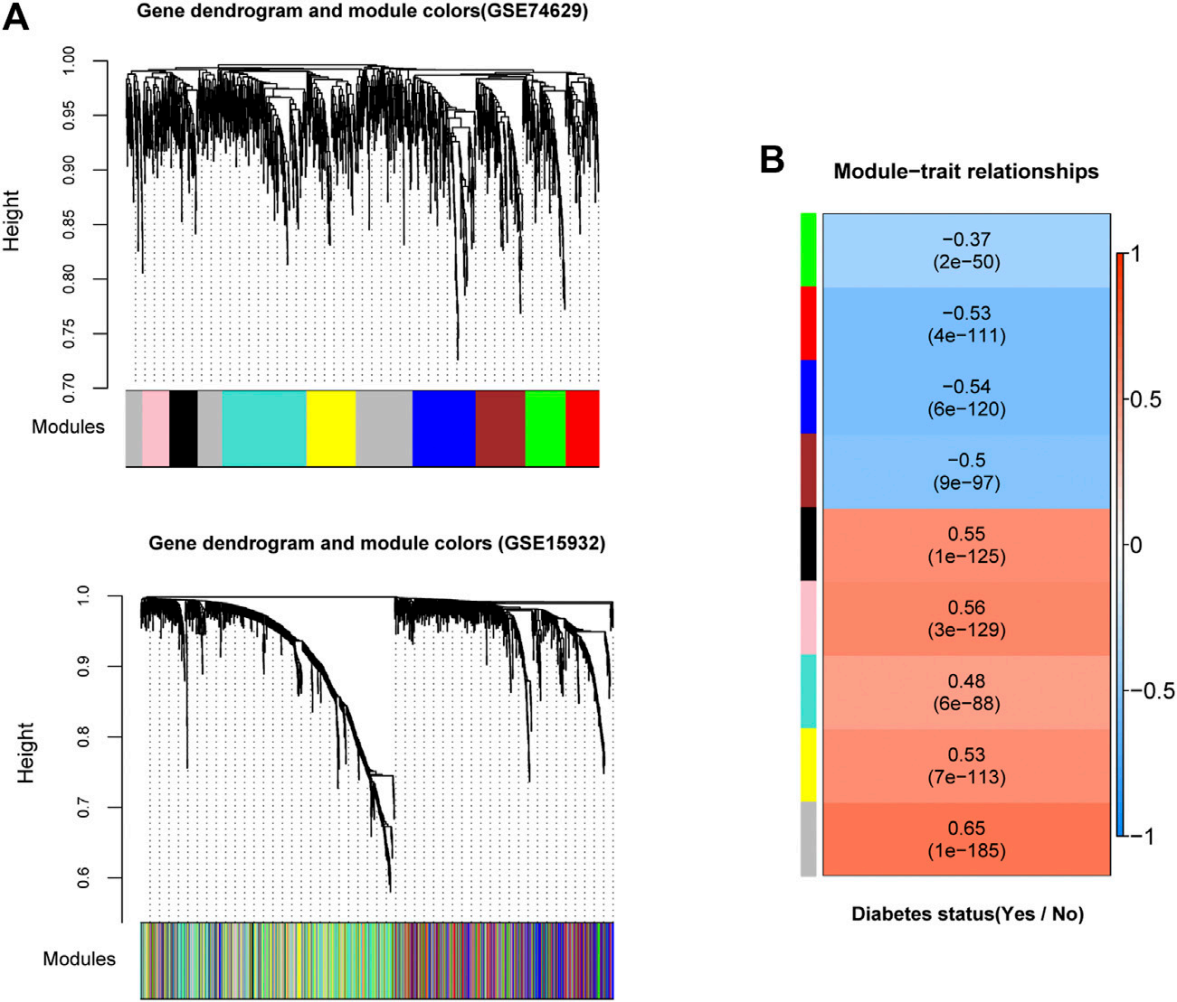
Identification of significant gene modules by weighed gene co-expression network analysis (WGCNA) **(A)**: The module partition in training and validation datasets (GSE74629 and GSE15932). Different colors represent different modules. For dataset GSE74629, nine gene modules (black, blue, brown, green, grey, pink, red, turquoise, and yellow modules) were identified to be associated with diabetes mellitus status **(B)**: The heatmap of correlations between the nine modules extracted from GSE74629 with diabetes mellitus status. The color of left side means different modules, and the color of right side ranged from blue to red means the correlation coefficient ranged from −1 to 1.

In order to examine whether gene expression levels in each dataset had comparability, we performed consistency analyses for the expressions of overlapping genes in two datasets. Results suggested that gene expression correlation and network node connection correlation were remarkably positive for training and validation sets (Supplementary Figure S2A). Notably, the scale-free network distribution was a prerequisite for WGCNA algorithm. Therefore, we calculated the square of the correlation coefficient (log(k) and log(p(k)) for the weight parameter of adjacency matrix (power parameter) under different values (Supplementary Figure S2B). We observed that the average connectivity of genes was 1 when power parameter was 10, indicating it has scale-free network characteristics (Supplementary Figure S2B). Herein, we obtained 9 gene modules associated with DM status by a co-expression network analysis with training set GSE74629 (Figure 2A). Meanwhile, module division was also carried out in validation set (GSE15932) as displayed in Figure 2A. The correlation analysis between gene modules and DM status was showed in Figure 2B. Four gene modules exhibited negative correlations with DM status (correlation coefficient <0) while five modules had a positive correlation with DM status (correlation coefficient >0). Finally, we evaluated the stability of gene modules. In general, a higher value of preservation Z score represents better module stability. More specifically, the module was stable with 5 < Z score <10 while the module had a good robustness with Z score >10. Our results demonstrated that blue and turquoise modules showed a good stability with preservation Z score >5 and *p* value ≤0.05 (Table 1). Moreover, we found that blue module had a negative correlation with DM status while there was a positive correlation between turquoise module and DM status (Figure 2B). Thus, the genes in these two modules (206 genes in blue module and 275 genes in turquoise module) were used for the following analysis.

**TABLE 1:** Preservation evaluation between GSE74629 and GSE15932 with regarding to the nine gene modules extracted from GSE74629 by weighed gene co-expression network analysis (WGCNA).

ID	Color	Module size	Preservation		
			Z-score	Cor	*p* value
Module 1	black	93	1.792942	0.12	0.25
Module 2	blue	206	8.285475	0.31	5.80E-06
Module 3	brown	163	3.119733	0.08	0.31
Module 4	green	132	3.42939	0.26	0.0026
Module 5	grey	323	8.777289	-0.53	8.60E-25
Module 6	pink	88	2.10382	-0.06	0.58
Module 7	red	106	2.981962	0.16	0.1
Module 8	turquoise	275	12.063853	0.27	5.60E-06
Module 9	yellow	160	7.446963	0.071	0.37

In general, a higher value of preservation Z score represents better module stability. The module was considered as stable with 5 < Z score <10 while the module had a good robustness with Z score >10. Cor: gene expression correlation.

### PPI Network

**FIGURE 3:**
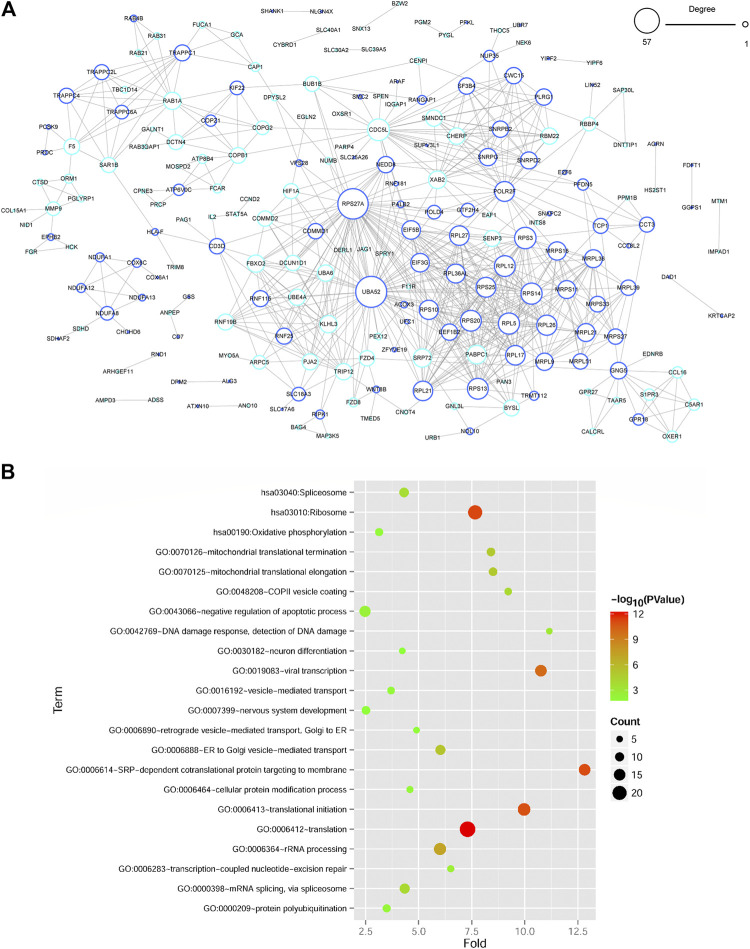
Protein-protein interaction (PPI) network construction and functional enrichment analyses of DEGs in PPI network **(A)** The PPI network of DEGs in two significant gene modules. The node size represents the degree of node, and the color of the edge of gene node shows the significant gene modules extracted by WGCNA. **(B)** Functional enrichment analyses of genes in PPI network. The vertical axis represents enrichment fold values and horizontal axis shows the names of GO-BP terms and KEGG pathways. Node size denotes the number of genes and the bigger node represents more genes. Node color indicates the enrichment significance, and the closer to the red the higher significance.

A PPI network of genes in blue and turquoise modules related to DM status was constructed based on STRING database. There were 214 gene nodes and 701 protein-protein interaction pairs (Figure 3A). Moreover, RPS27A (ribosomal protein S27a) and UBA52 (ubiquitin A-52 residue ribosomal protein fusion product 1) with a relatively higher degree were key genes in PPI network. Besides, functional analyses revealed that genes in PPI network were significantly related to 19 GO-BP terms, which were closely associated with translation-related terms, such as SRP-dependent cotranslational protein targeting to membrane and translational initiation process (Figure 3B, Supplementary Table S2). Meanwhile, three significant KEGG pathways were enriched for genes in PPI network, including ribosome, spliceosome, and oxidative phosphorylation OXPHOS pathways (Figure 3B, Supplementary Table S2).

### SVM Classification Analysis

**FIGURE 4:**
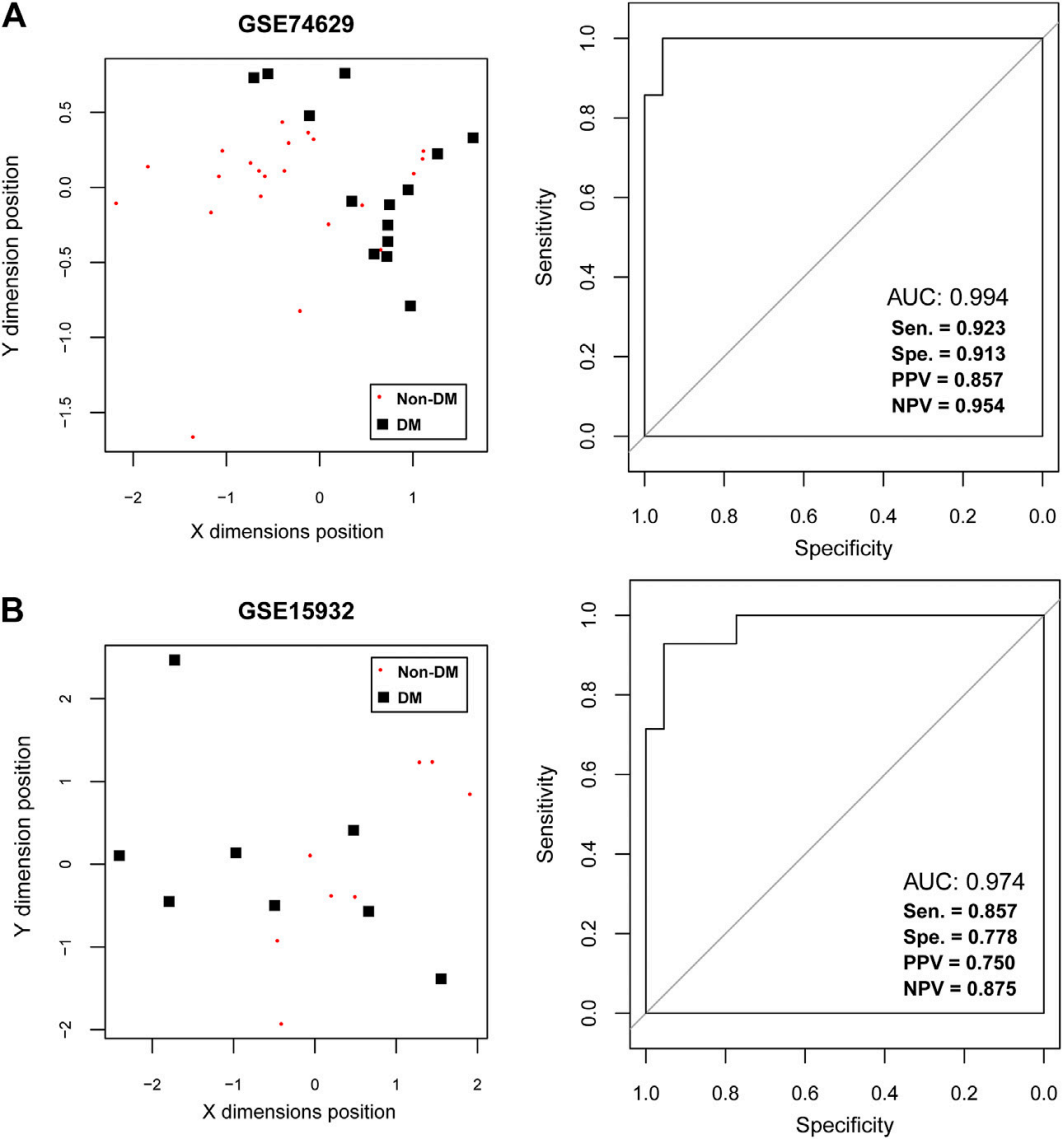
The support vector machine (SVM) classification analysis **(A)** The SVM analysis in training dataset GSE74629. The left figure shows the scatter plot of SVM classification based on 21 feature genes and right figure indicates the receiver operating characteristic curve of SVM classifier. **(B)** The SVM analysis in training dataset GSE15932. The left figure shows the scatter plot of SVM classification based on 21 feature genes and right figure indicates the receiver operating characteristic curve of SVM classifier. The black square represents the diabetic samples and red node represents the non-diabetic samples.

To further extract the optimal feature gene set, the number of feature genes in PPI network was reduced to 21 by RFE method with the max accuracy of 0.863 (Supplementary Figure S3 and Table 2). Notably, down-regulated *RPS27A* and *UBA52* were also belonged to the key feature gene set. After that, a SVM-based classifier was constructed with 21 feature genes in training and validation datasets to identify DM status (Figure 4). The performance evaluation of SVM classifier was then undertaken. Results suggested that SVM-based classifier significantly differentiated diabetic PDAC patients from non-diabetic PDAC patients in training set GSE74629 based on several assessment indicators (AUC = 0.994, sensitivity = 0.923, specificity = 0.913, PPV = 0.857, NPV = 0.954; Figure 4A). Furthermore, this classifier also could effectively distinguish diabetic and non-diabetic PDAC patients in validation set GSE15932 according to multiple assessment indexes (AUC = 0.974, sensitivity = 0.857, specificity = 0.778, PPV = 0.750, NPV = 0.875; Figure 4B). Taken together, our findings revealed that 21 feature genes had good discrimination ability for DM status and they might participate in the pathogenesis of diabetic PDAC.

**TABLE 2:** The list of 21 feature genes for diabetic-pancreatic ductal adenocarcinoma that identified by RFE method with max accuracy from the 214 genes in the protein-protein interaction (PPI) network.

Gene	*p* value	FDR	GSE15932-	Log2 FC	GSE74629-	Log2 FC
TRAPPC4	1.93E-04	0.008	down	−0.01614	down	−0.00508
SLC17A6	2.96E-06	0.000	down	−0.01824	down	−0.02077
TRAPPC6A	1.84E-04	0.007	down	−0.03159	down	−0.04112
KRTCAP2	2.23E-04	0.009	down	−0.02173	down	−0.00512
THOC5	4.43E-04	0.018	down	−0.01558	down	−0.05874
UBA52	1.58E-04	0.006	down	−0.01686	down	−0.03055
NUP35	1.12E-03	0.045	down	−0.07168	down	−0.01036
KIF22	3.05E-04	0.012	down	−0.02743	down	−0.03115
RPS27A	1.24E-04	0.005	down	−0.08765	down	−0.01318
ATP6V0C	1.16E-03	0.047	down	−0.05715	down	−0.02371
RPL26	6.81E-05	0.003	down	−0.02249	down	−0.02505
DPYSL2	1.62E-04	0.007	up	0.01931	up	0.01977
SLC39A5	2.03E-04	0.008	up	0.03015	up	0.03403
XAB2	3.08E-04	0.012	up	0.01209	up	0.05501
BZW2	1.63E-04	0.007	up	0.02933	up	0.00486
GPR27	1.06E-03	0.043	up	0.05109	up	0.00880
F11R	6.16E-04	0.025	up	0.01936	up	0.00782
DNTTIP1	3.57E-05	0.001	up	0.04530	up	0.07930
PYGL	1.19E-05	0.000	up	0.08198	up	0.02152
FBXO2	2.47E-04	0.010	up	0.01414	up	0.00816
SAP30L	1.95E-04	0.008	up	0.01824	up	0.03573

FC: fold change; FDR: false discovery rate.

### Survival Analysis

**FIGURE 5:**
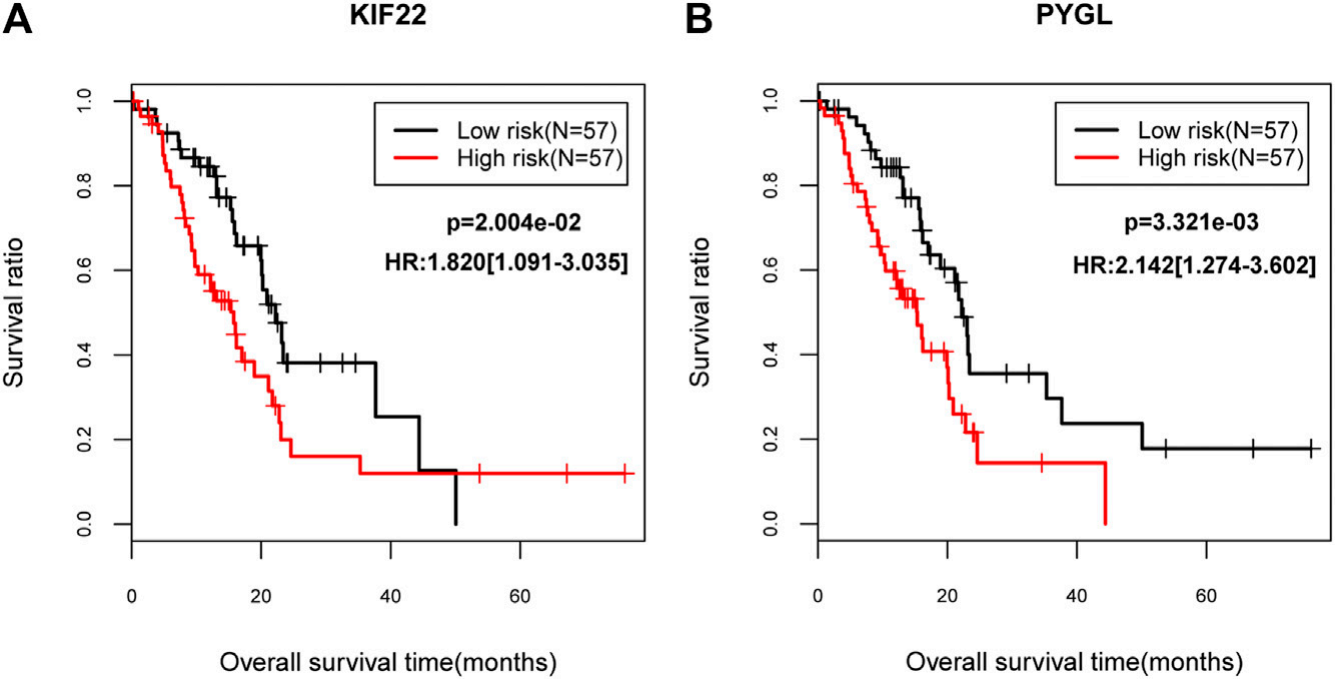
The Kaplan–Meier (KM) survival curves **(A)** The KM curve for KIF22. **(B)** The KM curve for PYGL. The black lines indicate the low risk group while the red lines indicate the high risk group.

Gene expression data of 150 PC patients were obtained. Totally, 114 patients had DM clinical information, of which, 33 subjects were diabetic PC patients, and 81 patients exhibited non-diabetic PC. SVM classification was verified by gene expression data from 114 patients and results suggested that this classifier could discriminate the diabetic from non-diabetic patients with PC based on multiple indicators (AUC = 0.924, sensitivity = 0.848, specificity = 0.938, PPV = 0.848, and NPV = 0.938; Supplementary Figure S4). In addition, univariate cox regression showed that down-regulated *KIF22* (kinesin family member 22) and up-regulated *PYGL* (glycogen phosphorylase L) were dramatically associated with prognosis of PC. Moreover, lower expression levels of *KIF22* (*p* = 2.004e-02) and *PYGL* (*p* = 3.321e-03) were strongly correlated with favorable survival outcomes according to the KM curves (Figure 5). Finally, the correlations between these two genes and other clinical factors were also evaluated. We found that *KIF22* was significantly associated with age, history of diabetes, alcohol history and neoplasm_histologic grade, while *PYGL* was markedly correlated with neoplasm_histologic grade (*p* < 0.05; Supplementary Table S3).

## Discussion

Many efforts have been made toward molecular genetics of PDAC over recent decades and researches have currently demonstrated that OXPHOS plays a central role in cancer cell energy provision rather than glycolysis [28–30]. For example, Ashton *et al* argued that OXPHOS level was up-regulated in several cancers, such as PDAC [31]. Viale *et al* found that surviving PDAC cells driven by *Ras* heavily relied on OXPHOS according to a transcriptomic and metabolic analysis [32]. Moreover, Zhou *et al* pointed out that inhibition of OXPHOS by drug metformin could increase apoptosis and induce cell cycle arrest in PDAC cells [33]. Herein, we performed functional enrichment analyses for genes in two gene modules associated with DM and found that many genes were significantly enriched in mitochondrial OXPHOS pathway, implying that OXPHOS might be implicated with the pathological mechanism of diabetic PDAC. Notably, the progression of DM was predominately related to the accumulation of damaged mitochondria in pancreatic β cells which secreted sufficient amounts of insulin [34]. Recently, Haythorne *et al* also emphasized that DM could trigger metabolic changes in pancreatic β-cells, such as remarkable reduction of OXPHOS-correlated pathways [35]. Therefore, detailed roles of OXPHOS in the development of diabetic PDAC still need to be elaborated in the future.

In this study, we extracted 21 feature genes and established a SVM-based classifier which had a good discrimination ability between diabetic PDAC and non-diabetic PDAC patients in training and validation datasets. Moreover, this classifier also could also differentiate diabetic PDAC from non-diabetic PDAC patients in an external dataset extracted from TCGA database. These genes might participate in the progression of diabetic PDAC. Moreover, we noted that two down-regulated feature genes (*RPS27A* and *UBA52*) with a higher degree are key genes according to the constructed PPI network. *RPS27A*, a member of ribosomal protein S27AE family, is a component of riobosome 40S subunit and encodes the carboxy terminus of ubiquitin. Previous studies have indicated that *RPS27A* was up-regulated in several cancers, including colorectal and renal cancers [36, 37]. Moreover, *RPS27A* induced cells cycle arrest, enhanced cell proliferation and suppressed cell apoptosis possibly via multiple signaling pathways, such as p53 and BCL-2 signaling pathways [38]. Yang *et al* conducted a bioinformatics analysis by constructing a miRNA-Transcription Factor-mRNA network to identify important genes related to mesenchymal stem cells (MSCs) for diabetic nephropathy (DN) treatment [39]. They stated that *RPS27A* was regulated by *EIF3M* (eukaryotic translation initiation factor 3 subunit M) and there was a higher *RPS27A* level in monocytes after mesenchymal stem cells co-cultured, suggesting *RPS27A* might play a critical role in the treatment of MSCs for DN [39]. However, few reports illuminated the potential roles of *RPS27A* on diabetic PDAC progression. Interestingly, PPI analysis showed *RPS27A* was closely interacted with *UBA52*, which was a housekeeping gene and could encode an ubiquitin ribosomal fusion protein. Although overwhelming evidence has demonstrated that *UBA52* was probably responsible for the pathogenesis of DN, the influence of *UBA52* on diabetic PDAC has not been fully understood [40, 41].

Additionally, two feature genes (down-regulated *KIF22* and up-regulated *PYGL*) exhibited close associations with prognosis of PC. Furthermore, patients with lower expression levels of *KIF22* and *PYGL* had better survival outcomes for PC. *KIF22*, a member of kinesin-like DNA-binding family, could encode a microtubule-dependent molecular motor protein and was involved in cell mitosis process [42]. A previous research reported that *KIF22* was up-regulated in breast cancer and its inhibition could significantly suppress cell proliferation [43]. Zhang *et al* argued that KIF22 mRNA and protein levels were over-expressed in prostate cancer, and *KIF22* was not dramatically linked with clinical outcomes of prostate cancer [44]. Herein, we found that *KIF22* was significantly associated with history of diabetes. Therefore, we inferred that this gene may be a key gene biomarker in the development of diabetic PDAC. Additionally, *PYGL* was identified as a metastasis-associated metabolic gene in prostate cancer [45]. Until now, the possible influences of *PYGL* on diabetic PDAC have not been investigated.

There were some limitations in this study. Firstly, our findings suggested that OXPHOS pathway was strongly involved in the development of diabetic PDAC. However, the precise mechanisms have not been clarified**.** Secondly, the number of available samples in our study is relatively low, and a comprehensive bioinformatics analysis based on a larger sample size and relevant experimental assays still need to be carried out to verify our findings. Thirdly, more clinical and physiological features need to be included for a more comprehensive survival analysis.

In summary, our results showed that OXPHOS pathway might participate in the pathogenesis of diabetic PDAC. Moreover, a SVM classifier based on 21 feature genes was built and this classification model dramatically distinguished diabetic and non-diabetic PDAC patients. Additionally, *KIF22* and *PYGL* were potential prognostic genes for PDAC survival. However, more detailed bioinformatics analysis and corresponding experimental assays are still need to be undertaken in the future.

## Data Availability

The original contributions presented in the study are included in the article/Supplementary Material, further inquiries can be directed to the corresponding author.
