# Supplementary material for: Identification of Key Genes Involved in Pancreatic Ductal Adenocarcinoma with Diabetes Mellitus Based on Gene Expression Profiling Analysis
Source: Pathol Oncol Res. 2021 Apr 20;27:604730. doi: 10.3389/pore.2021.604730 (PMC8262175; doi:10.3389/pore.2021.604730)
Supplement: Supplementary file 1 [file Table1.docx]

**Table S1 The correlations between** **KIF22 and** **PYGL and clinical factors.**

| **Clinical factor** | **KIF22** | | **PYGL** | |
| --- | --- | --- | --- | --- |
|  | **P value** | **Cor** | **P value** | **Cor** |
| age | 0.00607 | 0.0128 | 0.158 | -0.0115 |
| gender | 0.444 | 0.0833 | 0.334 | -0.1812 |
| history_of_chronic_pancreatitis | 0.162 | 0.2703 | 0.819 | -0.0762 |
| history_of_diabetes | 0.0453 | 0.0895 | 0.0784 | 0.0565 |
| alcohol_history | 0.45 | 0.0897 | 0.296 | -0.2169 |
| neoplasm_histologic_grade | 0.0332 | 0.1727 | 0.0473 | 0.2776 |
| pathologic_M | 0.67 | -0.2624 | 0.252 | -0.9834 |
| pathologic_N | 0.531 | -0.0808 | 0.0519 | 0.4289 |
| pathologic_T | 0.49 | -0.0739 | 0.598 | 0.0973 |
| pathologic_stage | 0.108 | -0.2272 | 0.713 | -0.0901 |
