# Supplementary figures and images for: Identification of Key Genes Involved in Pancreatic Ductal Adenocarcinoma with Diabetes Mellitus Based on Gene Expression Profiling Analysis
Source: Pathol Oncol Res. 2021 Apr 20;27:604730. doi: 10.3389/pore.2021.604730 (PMC8262175; doi:10.3389/pore.2021.604730)

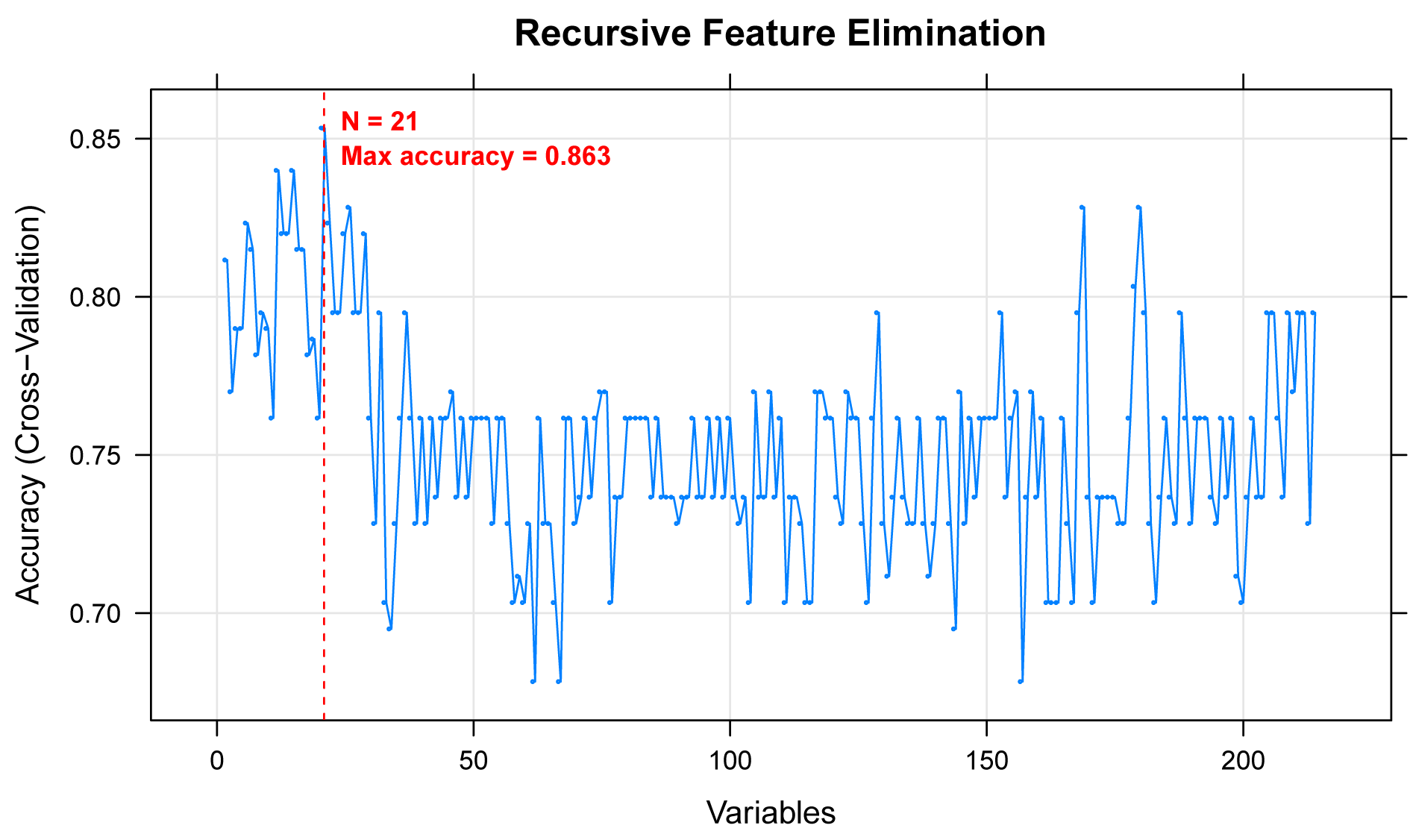

Supplement: Supplementary file 2 [file Image3.TIF]

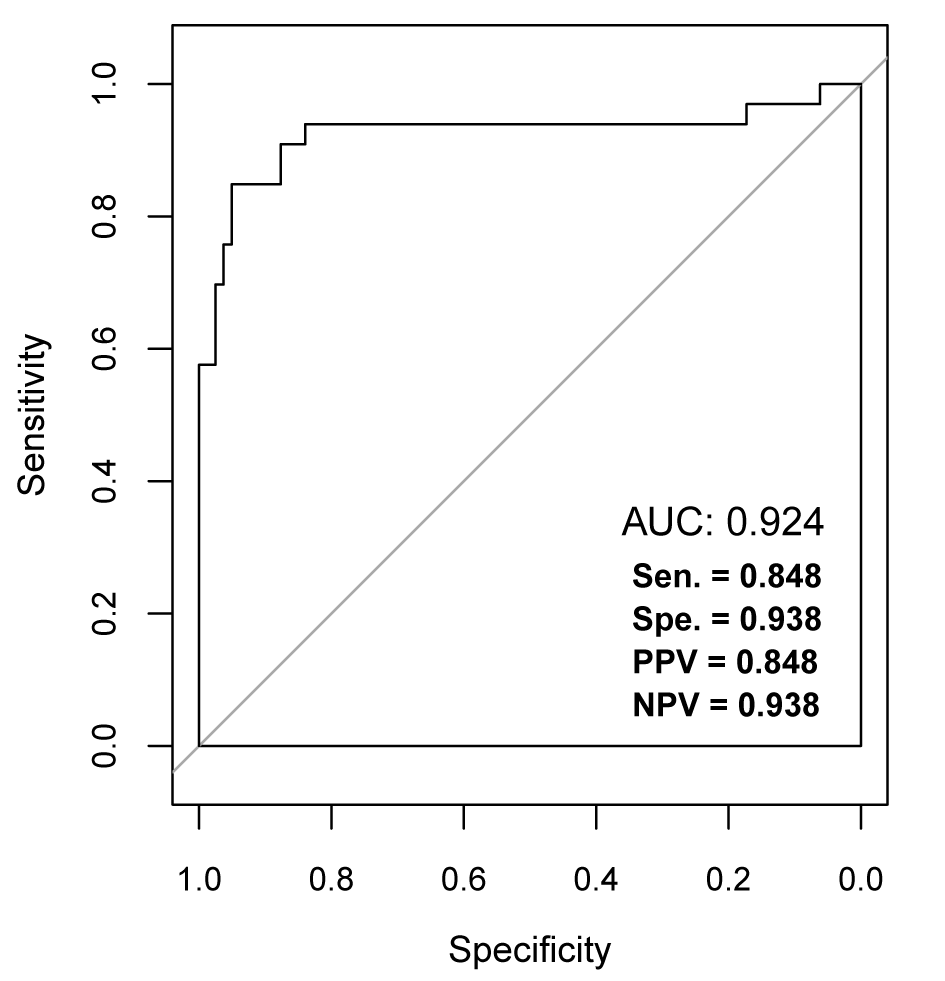

Supplement: Supplementary file 3 [file Image4.TIF]

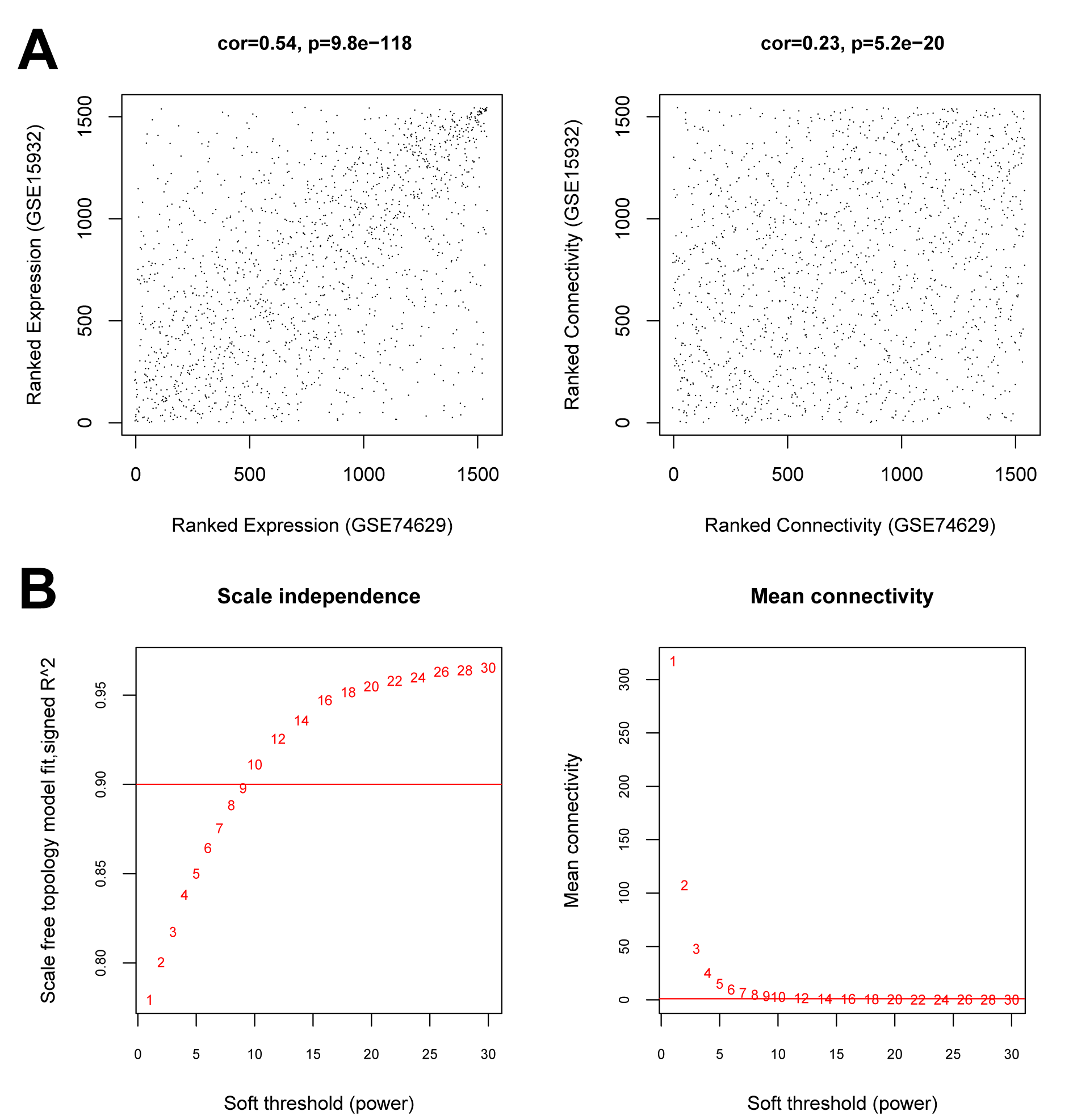

Supplement: Supplementary file 4 [file Image2.TIF]

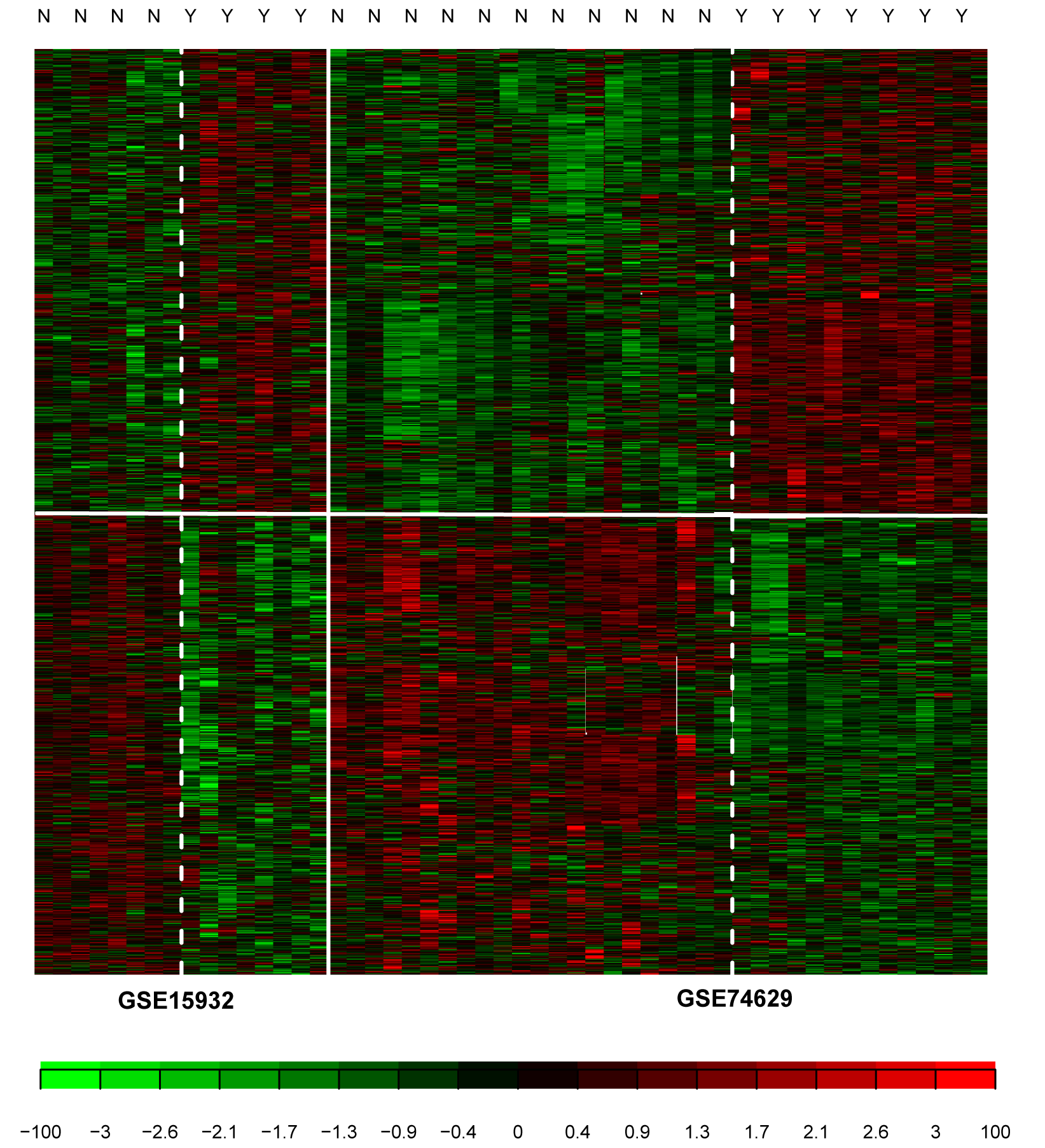

Supplement: Supplementary file 5 [file Image1.TIF]
